# Mitochondrial Long Non-Coding RNAs in Gynecological Cancers: Pathogenic Signaling Pathways and Therapeutic Opportunities

**DOI:** 10.3390/cimb48030261

**Published:** 2026-02-28

**Authors:** Ioana-Stefania Bostan, Nicolae Gica, Mirela Mihaila, Marinela Bostan, Nicoleta Radu, Viviana Roman, Cristina-Elena Dinu-Pirvu, Valentina Uivarosi

**Affiliations:** 1 Faculty of Medicine, “Carol Davila” University of Medicine and Pharmacy, 050471 Bucharest, Romania; ioana-stefania.bostan@rez.umfcd.ro (I.-S.B.); gica.nicolae@umfcd.ro (N.G.); 2 Filantropia Clinical Hospital, 011132 Bucharest, Romania; 3 Center of Immunology, Stefan S. Nicolau Institute of Virology, Romanian Academy, 030304 Bucharest, Romania; viviana.roman@virology.ro; 4 Faculty of Pharmacy, Titu Maiorescu University, 040314 Bucharest, Romania; 5 Department of Immunology, Victor Babes National Institute of Pathology, 050096 Bucharest, Romania; 6 Faculty of Biotechnologies, University of Agronomic Sciences and Veterinary Medicine of Bucharest, 59 Marasti Boulevard, District 1, 011464 Bucharest, Romania; nicoleta.radu@biotehnologii.usamv.ro; 7 Department of Biotechnologies, National Institute of Chemistry and Petrochemistry R&D of Bucharest, 202 Splaiul Independentei Street, District 6, 060021 Bucharest, Romania; 8 Department of Physical and Colloidal Chemistry, Faculty of Pharmacy, “Carol Davila” University of Medicine and Pharmacy, 6 Traian Vuia Str., 020956 Bucharest, Romania; cristina.dinu@umfcd.ro; 9 Innovative Therapeutic Structures Research and Development Centre (InnoTher), “Carol Davila” University of Medicine and Pharmacy, 6 Traian Vuia Str., 020956 Bucharest, Romania; 10 Department of General and Inorganic Chemistry, Faculty of Pharmacy, “Carol Davila” University of Medicine and Pharmacy, 6 Traian Vuia Str., 020956 Bucharest, Romania; valentina.uivarosi@umfcd.ro

**Keywords:** lncRNA, mitochondria, apoptosis, ovarian cancer, cervical cancer, endometrial cancer, chemoresistance, biomarkers

## Abstract

Understanding the complex molecular mechanisms behind gynecological cancers is crucial, as these diseases pose significant challenges to women’s health and are frequently diagnosed at advanced stages. Various genetic, epigenetic, and metabolic alterations play a vital role in tumor development, metastasis, and therapy. Exploring mitochondrial dysfunction and the role of lncRNAs may provide essential insights into how tumor cells evade apoptosis, alter their metabolic pathways, and adapt to stress. In gynecological malignancies, nuclear lncRNAs contribute to tumor progression, treatment resistance, and metastasis through mechanisms that include chromatin remodeling, microRNA modulation, and regulation of mitochondrial dynamics. More recently, the emerging role of mt-lncRNAs, derived from the mitochondrial genome, has attracted attention for their involvement in retrograde signaling, mitochondrial respiration, and regulation of apoptosis. Dysregulation of mt-ncRNAs may contribute to tumor bioenergetic reprogramming, mitochondrial integrity, and nuclear gene expression. The objective of this review is to consolidate the current understanding of the regulatory mechanisms of mitochondrial lncRNAs in ovarian, cervical, and endometrial cancers, thus identifying new opportunities of research. A thorough elucidation of the role of mitochondrial lncRNAs in mitochondrial–nuclear communication may facilitate the development of new interventions in gynecological oncology, highlighting the potential of these molecules as diagnostic biomarkers and therapeutic targets.

## 1. Introduction

Gynecological malignancies, which include ovarian, cervical, and endometrial cancers, represent a substantial global health challenge. These conditions are characterized by elevated morbidity and mortality rates, particularly in their advanced stages. Despite advancements in diagnostic and therapeutic approaches, the prognosis for numerous patients remains unfavorable, primarily due to late detection, recurrence of the disease, and resistance to conventional treatment options [1,2]. Statistical analyses of the global burden of gynecological cancers (ovarian, cervical, and endometrial) up to 2025 indicate 1,473,427 new cases and 680,372 deaths worldwide, representing about 15–16% of all newly diagnosed cancers and cancer-related deaths among women. Among those cancers, cervical cancer remains the most significant single contributor: nearly 45% of new gynecological cancer cases and over 50% of gynecological cancer-related deaths. The age-standardized incidence rate globally for gynecological cancers in 2022 was about 30.3 per 100,000 women, and the mortality rate was about 13.2 per 100,000 [3].

Gynecologic malignancies continue to cause significant morbidity and mortality among women worldwide, primarily due to inadequate early detection methods. Ovarian cancer illustrates this issue, with about 70% of cases diagnosed at advanced stages (FIGO III–IV), when treatment options are limited and five-year survival rates are low. This late-stage diagnosis results from both the biological diversity and often silent progression of these cancers, as well as the limited accuracy of current serum biomarkers. While Cancer Antigen 125 (CA125) and Human Epididymis Protein 4 (HE4), are useful for monitoring and risk assessment, they lack sensitivity for early-stage disease and specificity in distinguishing malignant from benign conditions, particularly in premenopausal women and those with inflammatory or endometriotic disorders [4]. Multivariate index tests, such as the Risk of Ovarian Malignancy Algorithm (ROMA), OVA1 Next Generation (Overa), and the Risk of Malignancy Index (RMI), improve diagnostic accuracy by integrating biochemical and clinical data but remain insufficient for population screening. However, these models are still inadequate for effective screening. Ongoing research focuses on incorporating novel molecular biomarkers, advanced analytics, and machine learning to enhance early detection, reduce unnecessary interventions, and improve survival outcomes [5]. Current data indicate that in uterine or ovarian carcinosarcomas, the median progression-free survival after the first relapse is around 4 months, with overall survival for advanced cases being less than 3 to 4 years despite aggressive treatment efforts [6]. Moreover, in recurrent endometrial cancer, while hormonal therapies show lower response rates of 10 to 20% and survival under one year, this highlights an area ripe for improvement. Although chemotherapy combinations can enhance response rates, they often do not lead to substantial increases in survival duration [7]. These insights underscore the pressing need for the development of earlier, more sensitive, and specific diagnostic technologies, as well as the exploration of innovative, personalized treatment strategies. Addressing these gaps is crucial for enhancing survival rates and improving the quality of life for patients affected by these complex diseases.

Mitochondria are mainly known for the production of ATP and the metabolism necessary to meet the bioenergetic and biosynthetic requirements of the cell [8]. Studies on tumor cells have highlighted the role of mitochondria in the generation of reactive oxygen species (ROS), participating to sustain a vicious cycle between oxidative stress, genomic instability and cancer development. Consequently, mitochondria are not only the “power houses” of the cell, but also specific sites for complex processes like oxidative phosphorylation (OXPHOS), ROS generation, apoptosis regulation (via cytochromec release, BAX/BCL2, etc.), metabolic flux (TCA cycle, glutaminolysis), and even mitochondrial–nuclear signaling [9].

Recent research has identified mitochondrial dysfunction as a hallmark of these cancers, linking altered oxidative metabolism, resistance to apoptosis, and redox imbalance to tumor aggressiveness [10,11]. Parallel to these discoveries, the functional relevance of long non-coding RNAs (lncRNAs) has gained increasing attention. lncRNAs are a class of RNA molecules longer than 200 nucleotides that, although they do not code for proteins, play an essential role in regulating gene expression. In recent years, studies on nuclear lncRNAs have significantly contributed to understanding the molecular mechanisms involved in cancer pathogenesis, revealing key roles in epigenetic, transcriptional regulation, and nuclear organization [12]. In gynecological malignancies—including ovarian, cervical, and endometrial cancers—nuclear-encoded lncRNAs such as *HOTAIR*, *MALAT1*, *PVT1*, and *H19* are involved in processes such as cell proliferation, tumor invasion, angiogenesis, and resistance to treatments [13,14]. All data suggest that lncRNAs can modulate gene expression through chromatin remodeling, transcriptional regulation, and post-transcriptional interactions with microRNAs and proteins [15]. Although research on nuclear lncRNAs has provided a solid foundation for understanding transcriptional regulation in cancer, exploration of mitochondrial-associated lncRNAs (mt-lncRNAs) offers new insight into how tumor cells modify their energetic function and survival mechanisms [16]. mt-lncRNAs are a subset of lncRNA molecules that localize to or interact with mitochondria, influencing their biogenesis, dynamics, and apoptosis control. Dysregulation of mt-lncRNAs may contribute to tumor bioenergetics reprogramming, mitochondrial integrity, and nuclear gene expression. While extensive research has been conducted on nuclear lncRNAs across various cancer types, including gynecological cancers [17,18], investigations into mt-lncRNAs remain in their early stages.

Mitochondrially encoded lncRNAs are an emerging and still incompletely characterized class of transcripts in cancer biology. Beyond SncmtRNA and ASncmtRNAs, transcripts such as lncND5, lncND6, and lncCytB are part of an expanding repertoire of mitochondria-derived noncoding transcripts that show differential expression or functional relevance in tumors. These molecules are becoming topics of increasing interest due to their roles in metabolic reprogramming, apoptosis, and tumor progression [19,20]. Given the fundamental role of mitochondria in energy metabolism, apoptosis, and oxidative stress-processes profoundly affected in cancer, the exploration of mt-lncRNAs is increasingly necessary.

Studies have shown that bidirectional communication between the nucleus and mitochondria is essential for maintaining cellular homeostasis. In this context, nuclear-lncRNAs (nu-lncRNA) may play a role in the nucleus-to-mitochondria anterograde regulation. At the same time, mt-lncRNAs can be involved in the mitochondria-to-nucleus retrograde regulation, which remains elusive for the moment. In cancer, this regulatory coordination can become dysregulated, leading to metabolic reprogramming, increased cellular survival, and resistance to therapeutic interventions [21]. The limited number of studies focusing specifically on mitochondrial long non-coding RNAs (mt-lncRNAs) and the mitochondrial–nuclear interactions governed by anterograde and retrograde signaling in gynecological cancers highlights the need for further exploration in this domain. Investigating metabolic complexity and intricate mechanisms of treatment resistance inherent in gynecological cancers could reveal critical insights that reshape the approaches to diagnosis, prognosis, and individualized treatment plans.

## 2. Nuclear lncRNAs Versus Mitochondrial lncRNAs

Nuclear lncRNAs are derived from the nuclear genome and possess the capability to function within either the nucleus or the cytoplasm. They play various cellular mechanisms, including the regulation of epigenetics, nuclear gene transcription, chromatin organization, and intracellular signaling [22].

Conversely, mitochondrial lncRNAs are a subcategory of long non-coding RNAs (lncRNAs) that are transcribed from the mitochondrial genome (mtDNA) and localized and active in mitochondria. They participate in the regulation of mitochondrial gene expression, energy homeostasis, and functions such as oxidative respiration, mitophagy, and ROS production. Although both lncRNAs can influence mitochondrial function, only mitochondrial lncRNAs are directly encoded by mitochondria. In contrast, nuclear lncRNAs can indirectly act on mitochondria or even be transported there, without having a mitochondrial origin [23]. The distinctions between nuclear lncRNAs and mitochondrial lncRNAs are grounded in their genetic origin, cellular localization, and the specific functions they fulfill, as delineated in Table 1.

Cancer cells alter their metabolism to support rapid growth and survive stress. Mitochondrial lncRNAs add a regulatory layer that links mitochondrial function to metabolic reprogramming and stress-response signaling in cancer. Table 2 provides a systematic summary of mt-lncRNAs associated with gynecologic malignancies and their proposed biological roles [24].

## 3. The Nuclear–Mitochondrial Regulatory Axis in Gynecologic Malignancies

The nuclear–mitochondrial regulatory axis refers to the dynamic, two-way communication between the cell nucleus and the mitochondria that coordinates gene expression, metabolism, bioenergetics, and stress responses [25].

**Figure 1:**
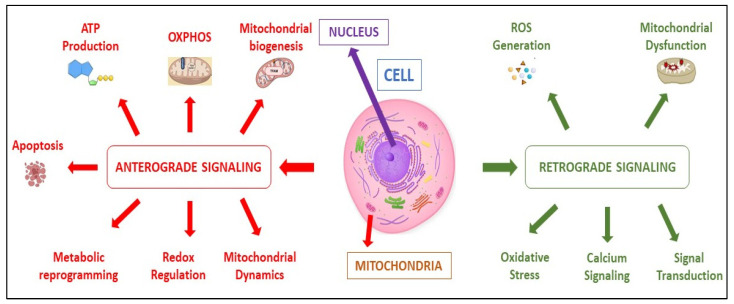
Mitochondria–Nucleus Crosstalk: Anterograde and Retrograde Signaling Pathways. Mitochondrial anterograde signaling involves dynamic crosstalk between the nucleus and mitochondria, regulating mitochondrial functions such as ATP production, oxidative phosphorylation (OXPHOS), biogenesis, metabolic reprogramming, mitochondrial dynamics, and apoptosis. In contrast, mitochondrial retrograde signaling refers to mitochondria-to-nucleus communication, transmitting mitochondrial stress signals, including reactive oxygen species (ROS), calcium flux, and dysfunction, to the nucleus to modulate cellular responses. These bidirectional signaling mechanisms collectively enable cellular adaptation to metabolic demands and stress conditions, whereas their dysregulation is associated with pathological states, including cancer.

In cancer, the nuclear–mitochondrial regulatory axis is pivotal in promoting tumor growth by facilitating metabolic reprogramming, enhancing stress resistance, evading programmed cell death, and inducing epigenetic modifications. Through bidirectional signaling, mitochondria assist the nucleus in activating pro-survival and pro-proliferative pathways, while the nucleus modulates mitochondrial function to meet cellular energy requirements, thereby promoting metastasis and contributing to resistance to therapeutic intervention [26] (Figure 1). The nuclear–mitochondrial communication axis plays a crucial role in cellular function through two well-coordinated signaling pathways. This pathway, known as anterograde signaling, flows from the nucleus to the mitochondria and plays a pivotal role in regulating mitochondrial function. It achieves this by controlling the expression of nuclear-encoded mitochondrial proteins and orchestrating mitochondrial biogenesis with the help of key transcriptional regulators such as Peroxisome proliferator-activated receptor gamma coactivator 1-alpha (PGC-1α), Nuclear factor erythroid-derived 2-like 1 & 2 (NRF1/2), and Mitochondrial Transcription Factor A, (TFAM). Furthermore, this pathway enhances metabolic programming, ensures redox balance, and fine-tunes mitochondrial dynamics, which includes processes like fusion, fission, and mitophagy [27].

The second pathway, retrograde signaling communicates vital information from the mitochondria back to the nucleus. This process responds effectively to changes in cellular energy balance, specifically the ratios of ATP, ADP, and AMP. It also activates the mitochondrial unfolded protein response (UPR^mt^) [28] and reacts to fluctuations in reactive oxygen species (ROS) and calcium levels, as well as variations in the availability of key metabolites like acetyl-CoA and NAD [29]. Additionally, it addresses any stress or damage to mitochondrial DNA. By sending these important retrograde signals, the mitochondria help reprogram nuclear gene expression, which is essential for maintaining cellular homeostasis, adapting metabolic pathways, and initiating robust stress-response mechanisms. This communication facilitates cellular responses to both internal and external challenges, thereby supporting overall cellular health and function.

### 3.1. Roles of the Mitochondrial Anterograde Signaling Pathway in Gynecological Cancers

Mitochondrial anterograde signaling (MAS) refers to the communication from the nucleus to mitochondria that regulates mitochondrial biogenesis, OXPHOS, metabolic pathways, mitochondrial dynamics, and stress responses [30,31]. This system is essential because mitochondria possess their own genome but rely heavily on nuclear-encoded proteins to maintain [32]. In tumor cells, which experience chronic metabolic stress, hypoxia, rapid proliferation, and altered nutrient availability, anterograde signaling is frequently rewired to optimize mitochondrial output. This allows tumor cells to generate ATP, maintain redox balance, and support biosynthetic pathways crucial for survival, growth, and metastasis [33]. Key molecular regulators of the MAS include PGC-1 coactivators, nuclear respiratory factors (NRFs), c-MYC, mTOR/AMPK pathways, estrogen-related receptors (ERRs), sirtuins, Hypoxia-Inducible Factors (HIFs), and components of the integrated stress response form an interconnected network orchestrating this nucleus-to-mitochondria communication [34].

In ovarian, endometrial, and cervical tumor cells, anterograde signaling pathways modulate cellular functions including proliferation, ROS buffering, mitophagy, apoptosis, and immune response (Table 3).

#### 3.1.1. MAS Pathways Control the Glycolysis–OXPHOS Balance in Tumor Cells

Tumor cells exhibit the ability to transition between glycolysis and OXPHOS in response to nutrient availability, oxygen levels, and environmental stressors (Table 3). This flexibility is controlled by a bidirectional communication mechanism between the nucleus and the mitochondria [35].

In the context of gynecological cancers, the regulation of mitochondrial biogenesis and OXPHOS is frequently disrupted. Nevertheless, tumor behavior is not uniform; tumor cells often adapt their mitochondrial programming in response to microenvironmental stresses, metastatic status, and specific oncogenic mutations [36]. The MAS pathway plays a central role in controlling OXPHOS capacity and enabling the metabolic flexibility required for tumor growth, survival, and therapy resistance [37].

The regulatory axis involves nuclear transcription factors and coactivators, such as PGC-1α, NRF1, NRF2, and TFAM, which coordinate the expression of nuclear-encoded mitochondrial proteins that form the electron transport chain (ETC) and regulate mitochondrial DNA replication and transcription. In contrast to normal cells, which maintain stable mitochondrial biogenesis through these factors, tumor cells display metabolic flexibility to adapt to changing energy demands [38]. Many primary ovarian, endometrial, and cervical tumors show suppressed PGC-1α signaling, favoring glycolysis and reduced mitochondrial activity to support rapid proliferation [39]. However, in metastatic or chemoresistant subpopulations, PGC-1α is frequently upregulated, driving enhanced mitochondrial biogenesis, elevated TFAM levels, increased mtDNA copy number, and strengthened ETC/OXPHOS capacity [40,41]. NRF1 and NRF2 become uncoupled from normal homeostatic control, with NRF2 acquiring additional antioxidant and pro-survival functions that help tumor cells tolerate oxidative and metabolic stress [42]. Collectively, these alterations produce a flexible mitochondrial program that allows gynecological tumor cells to switch between glycolytic and OXPHOS-dependent states, thereby supporting survival, invasion, and therapy resistance. For example, in cisplatin-resistant ovarian tumor cells, PGC-1α exhibits upregulation, which enhances mitochondrial biogenesis and OXPHOS, thereby facilitating survival in the presence of chemotherapeutic stress. Conversely, silencing of PGC-1α leads to a decline in mitochondrial function, an increase in lactate production, and heightened sensitivity to cisplatin [43,44]. Similarly, in high-grade serous ovarian cancer (HGSOC), increased levels of PGC-1α and TFAM proteins are linked to heightened expression of both nuclear- and mitochondrial-encoded OXPHOS subunits, highlighting the role of nuclear signaling in driving mitochondrial respiration in aggressive tumors [45]. Beyond biogenesis, PGC-1α, in conjunction with NRF1, regulates mitochondrial dynamics and quality control, including mitophagy. This occurs through induction of mitophagy receptors such as FUN14 domain-containing protein 1 (FUNDC1), a mitochondrial outer membrane protein that facilitates the removal of damaged mitochondria and preserves a robust, high-functioning mitochondrial population [46,47]. Supporting this, in type I endometrial cancer, increased expression of PGC-1α, NRF1, and TFAM correlates with elevated mitochondrial DNA content and citrate synthase activity, indicating enhanced mitochondrial mass and metabolic capacity [48].

In gynecological cancers, the MAS pathway activates NRF1 and NRF2, increasing the expression of electron transport chain (ETC) subunits. This enhancement improves mitochondrial respiration, providing the necessary energy for processes such as cell migration, invasion, and resistance to oxidative damage. Additionally, anterograde signals regulate the expression of metabolic genes involved in glycolysis, TCA cycle enzymes, fatty acid oxidation (FAO), and amino acid metabolism. Tumor cells in gynecological cancers can switch between glycolysis and oxidative phosphorylation (OXPHOS) depending on the stresses present in their microenvironment. For instance, FAO activation via PGC-1α is associated with metastatic cell survival, particularly in ovarian cancer. Furthermore, it appears that endometrial cancer cells utilize anterograde signaling to survive conditions of glucose deprivation. These mechanisms confer metabolic flexibility, allowing gynecological tumor cells to prefer OXPHOS during stressful situations, such as hypoxia or chemotherapy, while retaining the ability to revert to glycolysis when necessary. This adaptability is crucial for survival during metastasis, hypoxia, and nutrient deprivation.

#### 3.1.2. Regulatory Functions of MAS Pathways in Redox Balance, Antioxidant Protection, and Mitochondrial Structural Dynamics in Tumor Cells

MAS pathways play a critical regulatory role in maintaining redox homeostasis and orchestrating antioxidant defense mechanisms in gynecological cancers, where metabolic and oxidative stresses are often elevated due to rapid proliferation and an inflammatory tumor microenvironment (Table 3). Through coordinated communication from the nucleus to the mitochondria, these pathways regulate the expression of key antioxidant enzymes—including superoxide dismutases (SOD2), glutathione-related systems (GPX), catalase and thioredoxin system—thereby modulating ROS levels to support cancer cell survival. In gynecological malignancies such as ovarian, cervical, and endometrial cancers, dysregulation of anterograde signals enhances mitochondrial resilience, promotes metabolic reprogramming, and allows tumor cells to maintain a permissive redox state that favors proliferation, chemoresistance, and metastatic potential. By fine-tuning mitochondrial biogenesis, respiratory chain composition, and stress-response gene networks, anterograde signaling contributes to a redox balance that is not homeostatic in the normal sense, but strategically optimized to sustain malignant progression while avoiding cytotoxic ROS thresholds [49]. Some studies report that the mitochondrial deacetylase SIRT3, whose expression is regulated by nuclear transcriptional programs, controls SOD2 activity, H_2_O_2_-mediated signaling, and metabolic adaptation in ovarian cancer cells, including cells derived from ascites. This exemplifies how nuclear-directed (anterograde) signaling converges on mitochondrial antioxidant enzymes to regulate redox balance and tumor metabolism [50,51]. Another study indicates that NRF2 is the master antioxidant transcription factor, and in ovarian cancer, aberrant NRF2 activation increases antioxidant defenses and promotes chemoresistance. This links nucleus-driven antioxidant programs to tumor redox homeostasis [52]. Ma et al. demonstrated that activation of NRF2 promotes the expression of antioxidant genes and enhances cell survival in cervical cancer models. This provides an example from another gynecologic cancer showing that a nucleus-controlled antioxidant axis (NRF2 → antioxidant genes) protects tumor cells from oxidative stress and therapy [53]. Mitochondrial dynamics defined by the coordinated processes of fission and fusion and play an essential role in regulating cellular metabolism, redox balance, apoptosis, and adaptation to stress. These processes are controlled by a set of nuclear-encoded dynamin-related GTPases, including Dynamin-Related Protein 1 (DRP1, fission), outer-membrane fusion (MFN1/MFN2), and inner-membrane fusion and cristae remodeling (OPA1). In MAS, DRP1 and MFN2 are structural effectors of nuclear metabolic programs. In cancer, including gynecological malignancies these genes are transcriptionally and post-translationally regulated by nuclear oncogenic pathways that promote survival under metabolic and chemotherapeutic pressures [54]. In addition, experimental data demonstrate that PGC-1α not only promotes fusion gene expression but also represses DRP1 transcription under certain metabolic conditions, thereby shifting the dynamic equilibrium toward mitochondrial elongation in tumor cells. Conversely, nuclear oncogenic signaling may also enhance mitochondrial fission when fragmentation provides a selective advantage. The fission protein DRP1 is frequently activated via ERK1/2- or CDK1-mediated phosphorylation at Ser616, a modification driven by proliferative and stress-responsive nuclear programs. Increased DRP1 expression and activation facilitate mitochondrial segregation during rapid cell division, sustain glycolytic metabolism, and promote resistance to apoptosis. This plasticity—where fusion and fission pathways may both be upregulated—is characteristic of malignant cells undergoing metabolic reprogramming, enabling dynamic structural remodeling of the mitochondrial network according to environmental or therapeutic pressures [55].

In gynecological cancers, mitochondrial dynamics have emerged as critical determinants of chemoresistance, particularly in ovarian cancer, where mitochondrial function strongly influences cisplatin sensitivity. Studies in the SKOV3 cell line (ovarian cancer) model and its cisplatin-resistant derivative (SKOV3/DDP) show that resistant cells display a distinct mitochondrial phenotype characterized by downregulation of DRP1, upregulation of MFN2, elongation of mitochondria, and enhanced mitochondrial membrane potential. This shift toward a fused state reduces ROS accumulation during platinum-based treatment, maintains mitochondrial integrity, and attenuates activation of intrinsic apoptotic pathways [56]. Additional studies demonstrate that enhanced DRP1-mediated mitochondrial fission, or dysregulation of MFN2-dependent fusion, is observed in ovarian cancer models and can influence sensitivity to chemotherapeutic agents such as cisplatin. These findings link nuclear signaling pathways regulating mitochondrial fission–fusion machinery to alterations in mitochondrial morphology, reactive oxygen species (ROS) production, and therapeutic response [57]. By increasing the expression of genes related to mitochondrial fusion and controlling fission activity, nuclear programs help maintain stable metabolism, boost antioxidant defenses, and enable cells to adapt to chemotherapy injury. Understanding this communication provides valuable insights into the metabolic vulnerabilities of gynecological tumors.

#### 3.1.3. MAS Pathways as Key Regulators of Apoptotic and Cell Survival Programs in Tumor Cells

Anterograde signaling pathways, encompassing the molecular routes by which the nucleus regulates mitochondrial structure, function, and stress responses, constitute a central regulatory axis controlling apoptotic sensitivity and cell survival in gynecological cancers (Table 3). In ovarian, cervical, and endometrial malignancies, oncogenic nuclear programs-activated by hypoxia, DNA damage, nutrient stress, and chemotherapeutic exposure-modulate the transcription of genes involved in mitochondrial dynamics (DRP1, MFN1/2, OPA1), metabolic reprogramming (PGC-1α, NRF1/2), and apoptosis control (BCL-2 family proteins, caspases) [58]. These regulatory pathways converge on the mitochondria to dictate mitochondrial membrane integrity, ROS homeostasis, and cytochrome-c mobilization, thereby shaping the intrinsic apoptotic threshold of tumor cells [59].

In ovarian cancer, activation of nuclear HIF-1α and c-MYC pathways promotes the transcription of anti-apoptotic genes such as BCL-2 and MCL-1 while simultaneously inducing DRP1 activation, which maintains mitochondrial fragmentation and metabolic plasticity under hypoxia and chemotherapy. This nuclear-driven coordination of mitochondrial dynamics enhances cell survival by preventing mitochondrial outer-membrane permeabilization (MOMP) and limiting caspase-9 activation. Conversely, nuclear programs driven by PGC-1α and NRF1/2 favor mitochondrial fusion through the induction of MFN1/2 and OPA1, supporting stability, suppressing ROS generation, and providing resistance against genotoxic stress [60,61]. Studies in cisplatin-resistant ovarian cancer cells show that MFN2 overexpression or DRP1 inhibition reduces cytochrome-c release, maintains mitochondrial membrane potential, and suppresses apoptosis, thereby establishing mitochondrial fusion as a pro-survival phenotype under chemotherapy-induced metabolic stress. In cervical cancer models, MAS pathways mediated by FOXO3a and p53 regulate nuclear-encoded mitochondrial genes and transcriptional programs. This modulation of mitochondrial function directly affects the apoptotic machinery. Loss of p53 function—common in high-risk HPV-associated cervical carcinogenesis—disturbs anterograde control of BAX and PUMA transcription, impairing apoptotic competence and enhancing survival under oxidative and therapeutic stress. Endometrial cancers similarly exploit PGC-1α-mediated mitochondrial biogenesis and NRF2-driven antioxidant defenses to maintain redox balance and suppress intrinsic apoptosis. NRF2 hyperactivation increases transcription of antioxidant enzymes (SOD2, GPX4, PRDX3) that mitigate ROS accumulation, thereby preventing mitochondrial depolarization and caspase activation [62].

The MAS functions as a pivotal regulator of the mitochondrial apoptotic checkpoint in gynecological tumors. By coordinating mitochondrial metabolism, dynamics, antioxidant defense, and apoptotic protein expression, nuclear–mitochondrial communication enables tumor cells to evade programmed cell death and sustain growth under hostile microenvironmental and chemotherapeutic conditions. Targeting these pathways therefore represents a promising therapeutic strategy for overcoming chemoresistance and restoring apoptotic sensitivity in gynecological malignancies.

#### 3.1.4. MAS Pathways in Immune Modulation and Tumor Microenvironment (TME) Adaptation in Gynecological Cancers

MAS plays a central role in shaping the immune landscape and tumor microenvironment (TME) in gynecological cancers by coordinating transcriptional programs that govern metabolic plasticity, cytokine production, antigen presentation, and mitochondrial stress responses [63] (Table 2). In ovarian, cervical, and endometrial malignancies, nuclear transcription factors activated by hypoxia (HIF-1α), inflammation (NF-κB, STAT3), and metabolic stress (c-MYC, PGC-1α) control the expression of mitochondrial regulators that shape immunogenicity and TME adaptation, including metabolic enzymes like Lactate Dehydrogenase A (LDHA), Pyruvate Dehydrogenase Kinase 1 (PDHK1), mitochondrial biogenesis factors (NRF1/2), and stress-responsive genes such as Activating Transcription Factor 4 (ATF4), and C/EBP Homologous Protein (CHOP) [64,65]. These programs determine whether tumor cells adopt a predominantly glycolytic or oxidative phenotype, which in turn influences local oxygen availability, lactate accumulation, and pH gradients-key determinants of immune cell infiltration and function [66]. Mitochondrial stress signaling also intersects with nuclear programs to regulate the expression of immunomodulatory mediators such as PD-L1, IL-6, TGF-β, and VEGF, thereby shaping immune suppression, angiogenesis, and stromal remodeling within the TME [67,68]. For example, HIF-1α and NF-κB activation under hypoxic stress increases PD-L1 transcription in ovarian and cervical cancer cells, promoting T-cell exhaustion and immune evasion.

Tumor-associated macrophages (TAMs) contribute to MAS in cancer by activating nuclear transcriptional programs that regulate mitochondrial metabolism, redox balance, and stress responses. Transcription factors, such as HIF-1α, PPARγ/PGC-1β, NRF2, and STAT3, control mitochondrial biogenesis, oxidative phosphorylation, and fatty acid oxidation in TAMs, thereby shaping their functional polarization. In gynecological cancers, these mitochondrial programs favor immunosuppressive TAM phenotypes that alter the tumor microenvironment, facilitate immune evasion, and drive tumor progression and therapy resistance [69].

Moreover, anterograde signaling shapes tumor immune interactions by modulating antigen processing and mitochondrial apoptosis pathways. Transcriptional programs driven by ATF4, STAT3, and c-MYC suppress MHC-I expression, reduce antigenicity, and attenuate immunogenic cell death (ICD), thereby diminishing dendritic-cell activation and tumor recognition by CD8^+^ T cells (T cytotoxic) [70,71]. Simultaneously, nuclear control of mitochondrial permeability regulators, including BCL-2 family members and OPA1-decreases cytochrome-c release and blunts apoptosis-associated release of immunostimulatory signals such as ATP and mitochondrial DNA (mtDNA).

Because cytosolic and extracellular mtDNA activates cGAS–STING signaling, nuclear repression of pro-apoptotic mitochondrial pathways ultimately reduces type-I interferon production and diminishes T-cell recruitment into the TME [72,73]. MAS suppresses the immune response through the reprogramming of tumor metabolism and modification of the metabolic microenvironment, rather than by directly inducing cytokine-driven inflammatory signaling. Current evidence provide from preclinical and mechanistic studies indicates that anterograde signaling coordinates metabolic reprogramming, immunomodulatory gene expression, mitochondrial stress responses, and cell-death pathways to orchestrate TME adaptation and immune escape in gynecological cancers. Based on these experimental findings, targeting nuclear–mitochondrial regulatory axes has been proposed as a promising therapeutic strategy to restore antitumor immunity, enhance T-cell infiltration, and potentially improve the effectiveness of immunotherapies in gynecological malignancies. However, the majority of supporting data remain preclinical, and further clinical validation is required.

### 3.2. Roles of the Mitochondrial Retrograde Signaling (MRS) Pathway in Gynecological Cancers

Mitochondria are not just energy producers; they act as cellular sensors and signaling hubs. When mitochondrial function is perturbed, due to stress, mutations, hypoxia, or therapy, mitochondria transmit signals to the nucleus to alter gene expression. This MRS enables tumor cells to adapt metabolism, survive stress, modulate inflammation, and resist therapies [74].

MRS enables gynecological cancer cells to sense internal stress and communicate with the nucleus to adapt transcriptional programs. By modulating ROS, ATP/ADP, Ca^2+^, metabolites, and mtDNA integrity, mitochondria promote retrograde signaling, a key mechanism in tumor progression and chemoresistance, particularly in ovarian, endometrial, and cervical cancers [75]. The major functions and impacts of MRS in gynecological cancers are summarized in Table 4.

#### 3.2.1. MRS Pathways Role in Metabolic Reprogramming of Tumor Cells

Metabolic reprogramming is a hallmark of cancer, enabling tumor cells to sustain proliferation, resist stress, and adapt to environmental changes. Gynecological malignancies are highly dependent on metabolic flexibility, particularly because they frequently reside in hypoxic, nutrient-deprived microenvironments, such as the peritoneal cavity or inflamed endometrial tissue. MRS is a key regulatory pathway governing metabolic plasticity in gynecological malignancies, including ovarian, endometrial, and cervical cancers, by integrating mitochondrial dysfunction-derived signals with nuclear transcriptional programs. Metabolic flexibility refers to a tumor cell’s capacity to switch between oxidative phosphorylation (OXPHOS), glycolysis, and alternative substrate use, such as glutamine or fatty acids, in response to environmental stresses, including hypoxia, nutrient deprivation, or chemotherapeutic pressure [76] (Table 3).

Mitochondrial dysfunction induced by hypoxia, mtDNA mutations, oncogene activation, or chemotherapy activates retrograde pathways involving calcium-calcineurin-NFAT/NF-κB signaling, ROS-mediated stabilization of HIF-1α and NRF2, and integrated stress response factors such as ATF4 [77]. In gynecological cancers, these pathways interact with oncogenic landscapes—such as TP53 mutations in ovarian cancer, PI3K/AKT hyperactivation in endometrial cancer, and HPV E6/E7 expression in cervical cancer—amplifying mitochondrial stress responses and metabolic plasticity [78,79]. In ovarian cancer, MRS contributes to the HIF-1α stabilization under conditions of mitochondrial dysfunction and hypoxia, promoting glycolytic reprogramming that enables tumor cells to thrive within hypoxic peritoneal metastases. Concurrently, Ca^2+^-dependent stress signaling pathways, including calcineurin-NF-κB activation, support tumor cell survival and lipid metabolic programs required for colonization of the adipocyte-rich omentum. Together, these retrograde transcriptional responses enhance the expression of glycolytic enzymes, glutamine transporters, and fatty acid oxidation regulators, allowing ovarian cancer cells to dynamically shift between metabolic states in response to fluctuating oxygen and nutrient availability [80,81,82]. Similarly, in endometrial cancer, obesity-associated mitochondrial stress activates ATF4- and NRF2-dependent antioxidant and amino-acid metabolic programs, enhancing adaptability to oxidative stress and radiotherapy [83,84]. Cervical cancer cells with HPV oncogene-induced mitochondrial disruption use retrograde signaling to increase glycolysis, maintain redox balance, and support nucleotide biosynthesis required for rapid, virus-driven proliferation. Furthermore, MRS affects metabolic flexibility by driving epigenetic remodeling. Changes in mitochondrial metabolites, including α-ketoglutarate, acetyl-CoA, and NAD^+^, regulate histone and DNA modifications, which in turn adjust the expression of metabolic and survival genes [85,86,87]. These mitonuclear feedback loops sustain cancer cell stemness, promote resistance to chemotherapy (e.g., platinum drugs and PARP inhibitors), and enable adaptation to fluctuating microenvironmental pressures [88,89].

#### 3.2.2. Regulatory Functions of MRS Pathways in Redox and Calcium Homeostasis, Mitochondrial Biogenesis & Dynamics in Tumor Cells

MRS enables cancer cells to adapt to mitochondrial dysfunction by reshaping redox balance, calcium dynamics, and mitochondrial mass/structure [90,91]. Mitochondrial dysfunction leads to increased reactive oxygen species (ROS) release, which acts both as damaging agents and signaling molecules. MRS activates a set of antioxidant and prosurvival transcriptional responses that allow tumor cells to tolerate oxidative stress. Retrograde signaling promotes NRF2-, ATF4-, and NF-κB-driven antioxidant programs, leading to increased expression of SOD2, catalase, and components of the glutathione system, which are particularly important for ovarian cancer survival in oxidative microenvironments [92] (Table 4). Moderate ROS levels generated by dysfunctional mitochondria further stabilize HIF-1α, promoting glycolytic reprogramming, a feature frequently observed in cervical and ovarian cancers [93]. In endometrial carcinoma, ROS-induced NF-κB activation stimulates pro-inflammatory cytokines such as IL-6, amplifying tumor-promoting inflammatory loops [94]. Collectively, these redox adaptations support metastasis, enhance stemness, and confer resistance to platinum and taxane therapies [95].

Calcium (Ca^2+^) functions as a central mitochondrial retrograde signal linking mitochondrial stress to nuclear transcriptional reprogramming. Dysfunction at mitochondria–endoplasmic reticulum (ER) contact sites, known as mitochondria-associated membranes (MAMs), disrupts Ca^2+^ flux, leading to activation of Ca^2+^-dependent signaling pathways. These include calcium/calmodulin-dependent protein kinase (CaMK), calcineurin, nuclear factor of activated T cells (NFAT), and cAMP response element-binding protein (CREB), which collectively drive transcriptional programs that promote tumor cell survival and metabolic flexibility [96] (Table 4). When Ca^2+^ transfer from the ER to mitochondria is compromised, mitochondrial Ca^2+^ uptake decreases. This reduction inhibits the activation of Ca^2+^-sensitive enzymes within the tricarboxylic acid (TCA) cycle and limits the pro-apoptotic signaling that is typically initiated by mitochondrial Ca^2+^ overload. Consequently, the mitochondrial pathway of cell death is substantially weakened [97]. This mechanism is exploited in ovarian and cervical cancer cells, when the Ca^2+^ flux from ER to mitochondria is impaired—for example, due to reduced ER Ca^2+^ store content, altered expression or function of Ca^2+^-transport proteins (such as inositol-1,4,5-trisphosphate receptors, IP_3_Rs) or increased physical distance at MAMs—mitochondria fail to reach the Ca^2+^ threshold required to trigger apoptosis pathways. As a result, mitochondrial Ca^2+^ overload is avoided, mPTP remains closed, cytochrome c is retained, and apoptosis is not initiated. This fosters a state of apoptotic resistance [98]. A reduction in mitochondrial uptake facilitates an increased concentration of Ca^2+^ within the cytosol. This accumulation activates various Ca^2+^-dependent kinases and phosphatases, including calmodulin-dependent kinases and calcineurin. Consequently, these signaling molecules trigger the transcription factors activation such as NFAT and CREB, initiating gene expression programs that promote cell survival, metabolic remodeling, and adaptive responses to stress. Numerous studies demonstrate that impaired calcium dynamics between the endoplasmic reticulum and mitochondria are prevalent across various cancer types, including gynecological cancers, allowing cells to evade apoptosis and sustain their proliferative capacity [99]. Furthermore, cytosolic Ca^2+^ oscillations also enhance cytoskeletal remodeling and cell motility, facilitating peritoneal dissemination of ovarian cancer [100]. These Ca^2+^-dependent transcriptional adaptations additionally support cancer stem cell maintenance and stress survival in nutrient-poor or hypoxic microenvironments. Modulation of Ca^2+^ signaling not only enhances resistance to stresses such as nutrient deprivation, oxidative injury, or chemotherapeutic challenge, but also promotes metabolic flexibility, facilitating shifts between glycolysis and oxidative phosphorylation and supporting biosynthetic demands. Altered Ca^2+^ signaling in gynecological cancers is associated with poor prognosis, resistance to therapy, and enhanced invasive potential. This observation underscores the clinical relevance and mechanistic implications of Ca^2+^ signaling changes in these malignancies [101].

MRS also coordinates broad adjustments in mitochondrial mass, quality control, and structural organization. This includes stimulation of the mitochondrial biogenesis program, mediated by transcriptional regulators such as PGC-1α, TFAM, and NRF1, which work together to enhance mitochondrial DNA replication, transcription, and organelle turnover. Upregulation of these biogenesis factors serves as a compensatory response to mitochondrial dysfunction, enabling cancer cells to sustain ATP production and biosynthetic output despite impaired oxidative capacity [102,103].

In parallel, mitochondrial dynamics encompassing fission, fusion, and mitophagy are deeply sensitive to retrograde signaling cues and become reprogrammed to meet the metabolic and survival demands of tumor cells. Fission, primarily governed by the GTPase DRP1, is frequently upregulated in ovarian cancer and other gynecologic tumors. Enhanced fission supports rapid tumor cell proliferation, facilitates mitochondrial redistribution during mitosis, and amplifies ROS production to activate pro-survival and pro-proliferative signaling pathways [104]. Fusion, mediated by outer membrane proteins MFN1 and MFN2 and the inner membrane GTPase OPA1, promotes the formation of elongated mitochondrial networks. This fused morphology increases respiratory efficiency, enhances nutrient flexibility, and provides cytoprotective buffering against apoptotic stimuli such as chemotherapeutic agents or detachment-induced cell death [105]. In MRS, DRP1 and MFN2 become modulators of stress sensing and retrograde transcriptional activation.

Mitophagy, orchestrated by the PINK1/Parkin pathway and reinforced by metabolic sensors such as AMPK, selectively removes dysfunctional or ROS-generating mitochondria. This quality-control mechanism prevents excessive oxidative stress and contributes to maintaining a balanced mitochondrial pool in gynecological tumors, enabling sustained proliferation under metabolic and environmental stress.

Collectively, these MRS-driven alterations in mitochondrial biogenesis, dynamics, and quality control enhance the metabolic flexibility of gynecologic cancer cells. They help maintain a population of functional mitochondria capable of withstanding genotoxic injury and oxidative stress, thereby contributing directly to anoikis resistance during metastatic dissemination, survival in nutrient-poor microenvironments, and broad chemoresistance phenotypes. These data indicate that MRS plays a significant role in regulating the mechanisms through which gynecological cancers translate metabolic stress into pro-tumorigenic transcriptional programs. Through its influence on redox balance, calcium homeostasis, and mitochondrial remodeling, MRS contributes to tumor adaptation and survival under stress conditions.

#### 3.2.3. Retrograde Signaling Pathways as Key Regulators of Apoptotic and Cell Survival Programs in Tumor Cells

In gynecological cancers, mitochondrial dysfunction often activates retrograde signaling pathways, leading to changes in gene expression. Such mechanisms are critical in determining whether mitochondrial damage leads to apoptosis or initiates compensatory survival responses (Table 3).

Dysfunction in the mitochondrial electron transport chain leads to an increase in intracellular ROS. These ROS act as second messengers, connecting mitochondrial stress to nuclear transcriptional programs. Increased ROS levels activate stress-responsive kinases, such as JNK, p38 MAPK, and ERK. These kinases, in turn, orchestrate the regulation of pro-apoptotic transcription factors like c-Jun and p53, setting the stage for critical decisions about cell survival and death [106]. Some studies have shown that in gynecological cancers, ROS accumulation promotes intrinsic apoptosis by facilitating mitochondrial outer membrane permeabilization (MOMP), upregulating pro-apoptotic BCL-2 family proteins (e.g., BAX, BAK), and inducing cytochrome c release. Research in ovarian and cervical cancer models indicates that ROS-induced apoptotic priming increases sensitivity to chemotherapeutic agents, including cisplatin and paclitaxel [107,108]. A recent study highlights the crucial roles of ROS in the pathophysiology of endometrial cancer (EC). These molecules serve a dual purpose: they can promote tumor progression while also triggering apoptotic cell death. Furthermore, ROS influences cancer cell growth and the response to anticancer treatments by regulating the expression of specific genes [109]. However, cancer cells frequently counterbalance ROS signaling with antioxidant responses, highlighting the dual pro- and anti-apoptotic nature of ROS-mediated retrograde communication. Moreover, the disruption of mitochondrial membrane potentially alters cytosolic Ca^2+^ handling, leading to calcineurin activation, a Ca^2+^-dependent phosphatase. Calcineurin dephosphorylates transcription factors such as Nuclear Factor of Activated T cells (NFAT), Nuclear Factor kappa-light-chain-enhancer of activated B cells (NF-κB), and cAMP Response Element-Binding protein (CREB), enabling their nuclear translocation and regulation of genes involved in inflammation, metabolism, and apoptosis [110,111,112].

In the context of gynecological cancers, particularly ovarian carcinoma, activation of the calcineurin-NFAT signaling axis contributes to survival and therapy resistance. This phenomenon is largely attributed to the modulation of apoptotic regulators. Elevated cytosolic Ca^2+^ levels, which arise from mitochondrial stress, initiate the activation of calcineurin. This enzyme subsequently dephosphorylates NFAT family transcription factors, facilitating their translocation to the nucleus. Once in the nuclear environment, these transcription factors engage in transcriptional programs that enhance the expression of pro-survival genes while inhibiting apoptotic pathways. For example, NFATC4 nuclear translocation in ovarian cancer cells is induced by chemotherapeutic stress and is associated with quiescence and decreased sensitivity to cisplatin, indicating a role for calcineurin-NFAT in evading apoptosis in this context [113,114]. Activated NFAT can drive expression of genes such as c Myc and other survival factors, tipping the balance away from pro-apoptotic BH3-only proteins such as BIM, PUMA, and NOXA and reducing mitochondrial apoptotic signaling [115]. Concurrently, calcineurin activity promotes NF-κB signaling, which in ovarian cancer cells is well documented to foster anti-apoptotic and chemoresistant phenotypes by upregulating survival effectors (e.g., BCL-2 family proteins, XIAP) and repressing pro-apoptotic gene expression, thereby elevating apoptosis thresholds [116]. Through these integrated transcriptional networks, calcineurin-driven retrograde signaling promotes cell survival, stress adaptation, and resistance to therapy in ovarian and other gynecological malignancies.

Mitochondrial proteotoxic or oxidative stress activates the mitochondrial unfolded protein response (mtUPR), which transmits signals to the nucleus via transcription factors such as ATF4, ATF5 and (CHOP). These factors upregulate genes that restore proteostasis, enhance antioxidant defenses, and modulate apoptosis [117]. In the context of gynecological cancers, mtUPR and the broader integrated stress response influence apoptotic regulation through CHOP-mediated transcription of pro-apoptotic genes (e.g., PUMA, NOXA). Persistent or excessive mtUPR activation can trigger apoptotic collapse, particularly in cells with high mitochondrial dysfunction. Notably, in ovarian cancer, induction of ATF4 and CHOP correlates with increased sensitivity to mitochondrial-targeting therapeutics and ROS-inducing agents [118].

There are studies that have shown mitochondrial injury can lead to the release of mitochondrial DNA (mtDNA) into the cytosol, where it is recognized by the cGAS-STING pathway, a key component of innate immunity. cGAS binding to mtDNA generates cyclic GMP-AMP (cGAMP), which activates STING (Stimulator of Interferon Genes) and downstream transcription factors such as IRF3 and NF-κB, promoting type I interferon and inflammatory cytokine responses [119].

In the context of gynecological cancers, mtDNA-driven STING activation has dual consequences. Excessive mitochondrial stress can result in STING-mediated apoptosis, enhancing anti-tumor immunity, whereas chronic low-level STING activation may promote inflammation-associated tumor progression and immune evasion. Evidence from cervical cancer models shows that mtDNA stress amplifies apoptotic signaling through IRF3-dependent pathways, suggesting this retrograde mechanism contributes to tumor cell death under specific conditions.

#### 3.2.4. MRS Pathways in Immune Modulation and Tumor Microenvironment Adaptation in Gynecological Cancers

Tumor and stromal cells within the tumor microenvironment (TME) experience chronic stress from hypoxia, nutrient deprivation, and elevated ROS, necessitating extensive metabolic reprogramming (Table 4). Gynecological cancers commonly exhibit a Warburg-like phenotype characterized by enhanced aerobic glycolysis, while retaining functional OXPHOS, enabling metabolic plasticity that supports tumor growth, survival, and resistance to environmental and therapeutic stress.

MRS represents a critical regulatory mechanism underlying this adaptive capacity, conveying mitochondria-derived stress signals, including alterations in reactive oxygen species (ROS) levels, calcium (Ca^2+^) flux, and cellular energy imbalance, to modulate nuclear gene expression and influence immune cell function. Within the TME, these mitochondrial cues promote polarization of tumor-associated macrophages toward an immunosuppressive M2-like phenotype through ROS-, Ca^2+^-, and AMPK-dependent pathways, fostering angiogenesis, immune suppression, and tumor progression. Concurrently, persistent mitochondrial stress in tumor-infiltrating T cells impairs OXPHOS, increases ROS accumulation, and disrupts mitochondrial fitness, leading to T cell dysfunction and exhaustion and ultimately attenuating antitumor immunity [120,121]. MRS profoundly shapes immune cell function by integrating mitochondrial stress signals with immune regulatory pathways in the tumor microenvironment. Mitochondria-derived alterations in ROS levels influence antigen processing and presentation, modulate T-cell receptor signaling strength, and regulate cytokine production, thereby directly impacting immune activation and effector function [122]. In parallel, retrograde signaling interfaces with innate immune pathways such as STING and type I interferon signaling to regulate the expression of immune checkpoints, including Programmed Death-Ligand 1 (PD-L1), facilitating tumor immune escape [123,124,125]. Additionally, MRS reprograms the metabolism of both innate and adaptive immune cells, suppressing cytotoxic activity while favoring the expansion of immunoregulatory phenotypes. Tregs accumulate in the tumor microenvironment of ovarian, cervical, and vulvar cancers and suppress effector T cell and NK cell functions, promoting immune evasion and poor prognosis [126].

Gynecological cancers (ovarian, endometrial, cervical, vulvar) are characterized by a complex, heterocellular TME that includes cancer cells, immune infiltrates, fibroblasts, endothelial cells, and extracellular matrix components. This dynamic environment is shaped by metabolic stress and immune interactions that drive immune escape, angiogenesis, and metastatic progression In gynecological malignancies, where the tumor microenvironment is enriched with immunosuppressive cues, these mitochondrial-derived signals reinforce immune evasion by impairing effector T cell and natural killer cell function and promoting regulatory T cells and myeloid-derived suppressor cells, ultimately fostering tumor progression [127]. Although direct studies focusing on retrograde signaling in gynecological cancers are emerging, existing evidence suggests the TME in gynecological tumors exhibits marked immune suppression correlated with metabolic reprogramming and mitochondrial dysfunction, which may be mediated in part by retrograde signals [128]. Mitochondrial stress and retrograde signaling have been demonstrated to induce stem-like characteristics and facilitate epithelial–mesenchymal transition (EMT), a process associated with the emergence of cancer stem cells (CSCs). For example, in breast cancer models, mtDNA depletion activated calcineurin-dependent retrograde signaling, leading to EMT-like changes and the generation of cells with increased stemness characteristics [129]. Furthermore, MRS can directly influence the expression of stemness genes such as NANOG and SOX2, as well as CSC markers through mitochondrial-to-nuclear communication pathways that modify transcriptional networks [130].

MRS is activated in response to mitochondrial dysfunction and transduces stress-derived signals to the nucleus, inducing inflammatory and stress-responsive transcriptional programs. In cancer, this pathway can substantially remodel the immune microenvironment by regulating the expression of cytokines, chemokines, and other mediators that influence the recruitment, differentiation, and effector function of immune cells. Although evidence increasingly supports a role for MRS in tumor-associated inflammation, direct mechanistic studies linking specific MRS pathways to immune modulation in gynecological cancers are limited and require further investigation.

### 3.3. Comparative Roles of MAS and MRS in Cancer

Mitochondrial–nuclear communication occurs in two directions, MAS and MRS, each with distinct but complementary roles in cancer cell metabolism and therapy resistance. MAS establishes the basal metabolic and bioenergetic state in response to oncogenic signals and environmental factors, shaping intrinsic metabolic phenotypes, hypoxia responses, and baseline resistance. In contrast, MRS is activated by mitochondrial dysfunction, oxidative stress, or electron transport chain impairment, triggering nuclear transcriptional changes that support metabolic flexibility, glycolytic switching, hypoxia adaptation, and acquired chemoresistance. MAS defines the baseline metabolic identity, while MRS enables adaptive responses to cellular stress and therapeutic challenges (Table 5). Both signaling pathways regulate redox balance, antioxidant defenses, and mitochondrial dynamics. MAS sets the baseline mitochondrial structure and redox state under oncogenic control, whereas MRS rapidly activates antioxidant defenses, modulates DRP1/MFN2-driven remodeling, and controls ROS to maintain mitochondrial integrity and support tumor cell survival. In cancer, MAS indirectly suppresses anti-tumor immunity by altering tumor metabolism and creating a metabolically restrictive microenvironment. In contrast, MRS more directly influences immune responses through mitochondrial stress-induced inflammatory signaling.

Although MAS and MRS frequently involve overlapping transcriptional regulators, such as HIF-1α, NRF2, and PGC-1α, the functional roles of these factors are highly context-dependent and determined by the directionality of signaling. Within MAS, HIF-1α drives glycolytic reprogramming and suppresses oxidative phosphorylation as part of a programmed adaptive response to hypoxic stress. In parallel, NRF2 maintains basal antioxidant defenses under oncogenic control, while PGC-1α promotes mitochondrial biogenesis and specifies metabolic phenotypes to support sustained tumor growth. In contrast, during MRS, these same regulators are activated in response to intrinsic mitochondrial stress signals. Under these conditions, HIF-1α is stabilized by mitochondrial reactive oxygen species or respiratory dysfunction to induce emergency metabolic reprogramming, NRF2 mediates acute redox defense and survival pathways, and PGC-1α facilitates compensatory mitochondrial repair and adaptive remodeling. Collectively, these bidirectional signaling mechanisms enable cancer cells to integrate preprogrammed metabolic states with stress-induced adaptive responses, thereby supporting tumor progression and contributing to therapeutic resistance [131].

It is important to note that the same transcription factors (HIF-1α, NRF2, PGC-1α, etc.) are often involved in both anterograde and retrograde mitochondrial signaling, but their modes of activation and biological outcomes differ substantially (Table 6).

## 4. The Therapeutic Potential of Mitochondrial lncRNA in Cancer

Although substantial progress has been made in surgical, chemotherapeutic, and targeted treatment strategies, late-stage diagnosis and the development of therapeutic resistance continue to significantly limit survival outcomes in aggressive gynecological cancer subtypes, which are associated with poor prognosis and frequent recurrence. In recent years, long noncoding RNAs (lncRNAs), have emerged as key regulators of cancer biology, influencing gene expression, cellular metabolism, proliferation, metastasis, and response to therapy. Within this broader class, mitochondrial lncRNAs, which either originate from mitochondrial DNA or localize to mitochondria to interact with mitochondrial and nuclear signaling pathways, are gaining recognition for their roles in modulating tumor metabolism, apoptotic signaling, and cellular homeostasis—processes critically altered in cancer cells [132].

Despite the limited functional characterization of mitochondrial lncRNAs in gynecological cancers, increasing evidence indicates that both mtDNA-encoded and nuclear-encoded mitochondrial lncRNAs are involved in anterograde and retrograde signaling pathways (Table 7).

### Therapeutic Targeting/Emerging Strategies

Mitochondria play a central role in tumorigenesis by regulating bioenergetics, redox homeostasis, and apoptotic signaling. Non-coding RNAs encoded in the mitochondrial genome, as well as nuclear non-coding RNAs that localize to or functionally interact with mitochondria in malignancies, including gynecological diseases, represent an attractive and relatively unexplored therapeutic target [133]. Recent studies and an increasing interest within the scientific community in targeting mitochondrial lncRNAs as a therapeutic approach for cancer are advancing this concept toward practical application. However, it is important to note that the majority of research remains in the preclinical and exploratory phases, particularly concerning gynecological cancers (Table 8).

Strategies for targeting mt-lncRNAs in cancer have evolved rapidly, focusing on both direct inhibition of mt-lncRNAs and blocking their indirect oncogenic functions (Table 5).

One of the best documented families of mitochondrial lncRNAs comprises the sense non-coding mitochondrial RNA (SncmtRNA) and the antisense non-coding mitochondrial RNAs (ASncmtRNA-1 and ASncmtRNA-2). These RNAs are transcribed from the mitochondrial genome and then exported to the cytosol and nucleus. SncmtRNA is expressed in both normal and cancer cells and plays a role in maintaining mitochondrial integrity and promoting cell proliferation. In contrast, ASncmtRNAs are selectively downregulated in tumor cells, including immortalized cervical cancer lines and other gynecologic tumor models, suggesting that loss of ASncmtRNAs may contribute to carcinogenesis (Table 7) [134].

**Figure 2:**
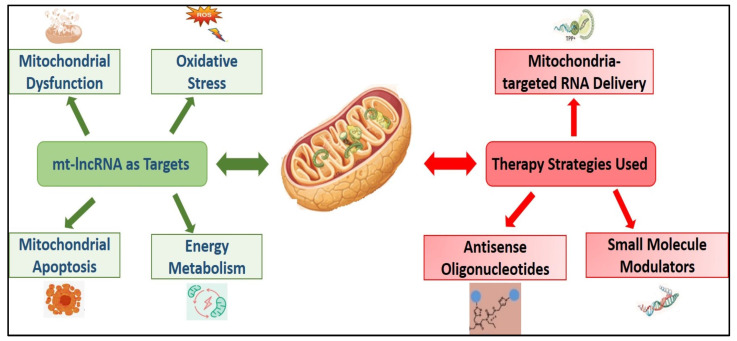
Therapeutic strategies targeting mitochondrial long non-coding RNAs (mt-lncRNAs) in cancer. Schematic representation of mitochondria-localized long non-coding RNAs (mt-lncRNAs) as emerging therapeutic targets in cancer. mt-lncRNAs regulate key mitochondrial functions, and their dysregulation of mt-lncRNAs contributes to mitochondrial dysfunction, metabolic reprogramming, and therapy resistance in tumor cells. Therapeutic strategies include mitochondria-targeted RNA delivery systems (e.g., nanoparticles or lipophilic cations such as triphenylphosphonium, TPP^+^), antisense oligonucleotides for lncRNA inhibition, small-molecule modulators of lncRNA–protein interactions, and ncRNA-based nanoformulations aimed at restoring mitochondrial apoptotic signaling and chemosensitivity.

ASncmtRNA-1 and ASncmtRNA-2 (Antisense mitochondrial lncRNAs) are downregulated in cervical, ovarian, and endometrial cancer. In cancer cells, loss of ASncmtRNAs is associated with increased proliferation and avoidance of apoptosis, facilitating tumor progression. In addition, ASncmtRNA 2 may serve as a precursor for certain miRNAs that regulate the expression of genes involved in the cell cycle, proliferation, and survival. Currently, the most advanced targeting strategy involves the use of antisense oligonucleotides (ASOs). Functional targeting of ASncmtRNAs with ASOs induces profound anti-tumor effects in preclinical models (Table 8). The knockdown of ASncmtRNAs triggers inhibition of cell proliferation, loss of mitochondrial membrane potential, caspase activation, and apoptotic cell death in multiple cancer cell types, while sparing normal cells. This effect is partly mediated by downregulation of survivin and other survival factors, demonstrating a tumor-selective vulnerability that is conserved across cancer types (Figure 2).

Preclinical evidence in different cancer models show that ASncmtRNA ASOs (e.g., Andes-1537S) reduce proliferation and induce apoptosis in bladder cancer cell lines, showing loss of mitochondrial membrane potential, Annexin V positivity, caspase-3 activation, and downregulation of survivin/Bcl-xL-again with minimal effects on normal cells [135]. Although direct studies in ovarian and cervical cancer models are limited, the differential expression of ASncmtRNAs in gynecologic tumors and their functional importance in bladder and other cancers support further exploration of ASO-based therapies in these settings [136]. ASncmtRNA 1 and ASncmtRNA 2 function as tumor suppressors and selective therapeutic targets, with potential applicability in multiple cancer types. Consequently, this approach has the advantage of high selectivity and low toxicity, offering a promising concept for the development of innovative anticancer therapies.

lncND5 is another mitochondrial genome-encoded long non-coding RNA derived from the ND5 locus, which encodes a core subunit of mitochondrial complex I. In cancer, the role of lncND5 remains poorly defined, and the evidence is much less extensive than for lncRNAs such as SAMMSON, H19, or LINC00152. Although direct functional studies in ovarian cancer are still limited, dysregulated expression of lncND5 has been reported in cancer-associated mitochondrial transcriptomic analyses and is thought to influence complex I activity, mitochondrial respiration, and metabolic adaptation [137]. Given that ovarian cancer frequently exhibits mitochondrial dysfunction and altered reliance on OXPHOS, lncND5 may contribute to tumor metabolic plasticity and disease progression [138]. Together with other mitochondrial lncRNAs, lncND5 represents a promising but underexplored regulatory layer linking mitochondrial gene expression to oncogenic metabolic reprogramming, warranting further functional investigation in gynecologic malignancies.

lncCYTB is a mitochondrial genome–encoded long non-coding RNA transcribed from the cytochrome b (CYTB) locus, which encodes a core component of respiratory complex III. In gynecological cancers, particularly ovarian and endometrial carcinoma, mitochondrial OXPHOS is critical for tumor progression and chemoresistance. While the specific function of lncCYTB remains uncharacterized, dysregulation of mitochondrial transcripts has been associated with changes in ETC activity and redox signaling in cancer cells. High-grade serous ovarian cancer (HGSOC) displays metabolic heterogeneity, with OXPHOS-dependent subpopulations linked to platinum resistance and poor prognosis [139]. As complex III is a major source of mitochondrial reactive oxygen species (ROS), altered regulation of CYTB-derived transcripts such as lncCYTB may affect ROS-mediated signaling pathways, including HIF-1α and NF-κB, as well as apoptosis sensitivity and adaptive stress responses. Increased mitochondrial respiration and ROS buffering capacity in chemoresistant ovarian tumors suggest that mitochondrial RNA regulators may contribute to metabolic plasticity (Table 7) [140].

In addition to mt-lncRNAs, several nuclear-encoded long non-coding RNAs (lncRNAs) have been shown to interact with mitochondrial pathways, thereby modulating cancer cell metabolism, survival, and proliferation. Among these, SAMMSON (Survival-Associated Mitochondrial Melanoma-Specific Oncogenic lncRNA) has been most extensively characterized in melanoma, yet emerging evidence indicates its functional relevance in other malignancies, including ovarian and breast cancer. SAMMSON exerts its oncogenic role primarily through direct interaction with the mitochondrial protein p32 (C1QBP), which is essential for proper 16S rRNA processing within mitochondria and for the oxidative phosphorylation maintenance. Disruption or loss of SAMMSON impairs mitochondrial protein synthesis, decreases mitochondrial membrane potential, and triggers apoptosis, underscoring its pivotal role in sustaining mitochondrial function and tumor cell viability [141]. The mechanistic involvement of SAMMSON in mitochondrial biogenesis and metabolic regulation suggests that it may contribute to tumorigenesis in many types of cancer, particularly in subtypes characterized by altered mitochondrial metabolism, such as ovarian or cervical cancer. Notably, SAMMSON has also been implicated in modulating chemosensitivity and metabolic orientation in doxorubicin-resistant breast cancer cells, highlighting its broader impact on cancer cell metabolic plasticity and therapeutic response [142,143].

Emerging delivery strategies may further enhance the translational potential of SAMMSON-targeted therapies. Advances in ASO chemistry, including phosphorothioate backbones and 2′-O-modified nucleotides, have been shown to enhance nuclease resistance, binding affinity, and intracellular uptake, thereby increasing target engagement and pharmacokinetic stability [144,145]. In addition to these chemical modifications, nanoparticle-based delivery systems such as lipid nanoparticles (LNPs), polymeric nanoparticles, and ligand-targeted liposomes offer further protection from enzymatic degradation. These approaches enable preferential accumulation in tumor tissue through enhanced permeability and retention effects or active targeting strategies [146,147]. Furthermore, such systems may allow co-delivery with chemotherapeutic agents, exploiting synthetic lethality in metabolically stressed tumor cells. Together, these delivery innovations may overcome key barriers to clinical translation by improving tumor specificity, reducing systemic toxicity, and enabling rational combination therapies.

Another nuclear-encoded lncRNA, LINC00152 (also known as CYTOR), is frequently upregulated in epithelial ovarian cancer and promotes tumor cell proliferation and survival. Its oncogenic activity is mediated in part by sequestering microRNAs that regulate pro-apoptotic factors, including MCL-1, which affects the mitochondrial apoptosis pathway by altering mitochondrial membrane stability. Although LINC00152 is not mitochondrially encoded, its ability to modulate apoptotic signaling through these pathways underscores an indirect but functionally relevant impact on mitochondrial regulation in gynecologic cancers [148,149].

While not strictly mitochondrial in origin, several additional lncRNAs implicated in gynecologic cancers, such as H19, have been linked to metabolic reprogramming and resistance to apoptosis. H19 contributes to chemoresistance and metabolic adaptation by modulating key signaling pathways that intersect with mitochondrial energy homeostasis, although the exact mechanisms remain under active investigation [150,151,152].

Growth Arrest-Specific 5 (GAS5) is a long non-coding RNA with well-established tumor-suppressive functions. In gynecological malignancies, including ovarian, cervical, and endometrial cancers, GAS5 expression is consistently downregulated and is associated with aggressive tumor behavior, therapy resistance, and poor clinical outcome. GAS5 regulates cancer progression through modulation of cell cycle arrest, apoptosis, and stress-response pathways, primarily by acting as a competing endogenous RNA for oncogenic microRNAs and by interfering with pro-survival signaling cascades [153,154].

In ovarian cancer, reduced GAS5 expression correlates with advanced stage, high tumor grade, and resistance to platinum-based chemotherapy. Functionally, GAS5 suppresses proliferation and invasion while promoting mitochondrial-mediated apoptosis through regulation of microRNAs such as miR-21 and miR-222 and downstream pathways including PI3K/AKT. Restoration of GAS5 expression enhances chemosensitivity, highlighting its potential as a therapeutic target [155]. In cervical cancer, GAS5 downregulation facilitates tumor growth and invasion via modulation of the miR-106b/PTEN axis and impaired intrinsic apoptotic signaling. GAS5 has also been implicated in HPV-associated metabolic reprogramming and mitochondrial dysfunction. In endometrial cancer, GAS5 loss is linked to hormone-dependent proliferation and reduced apoptotic capacity, suggesting a role in regulating hormone-responsive mitochondrial metabolism [156].

Recent studies indicate that a mitochondrial pool of GAS5 (mt-GAS5) contributes to intrinsic apoptosis and metabolic control. Loss of mt-GAS5 promotes mitochondrial dysfunction and apoptosis resistance, key features of treatment-refractory gynecological tumors. Therapeutic strategies aimed at restoring GAS5 levels, including lncRNA replacement, epigenetic reactivation, and mitochondria-targeted delivery systems, represent promising approaches to sensitize gynecological cancers to conventional therapies [157].

## 5. Conclusions and Future Perspectives

In conclusion, LncRNAs are key regulators of nuclear–mitochondrial communication in gynecological cancers, enabling metabolic flexibility, stress resistance, and oncogenic rewiring. Both nuclear-derived lncRNAs and mt-lncRNAs contribute to a coordinated signaling network that shapes tumor development and treatment response. Targeting this RNA-centered communication axis offers exciting opportunities for developing innovative therapies and precision biomarkers in gynecologic oncology.

Targeting mitochondrial genome-encoded long non-coding RNAs (mt-lncRNAs) represents a novel, conceptually strong, but still emerging therapeutic strategy in cancer biology, including gynecological malignancies. These lncRNAs, transcribed directly from mitochondrial DNA, are uniquely positioned to regulate core mitochondrial functions, including oxidative phosphorylation, metabolic reprogramming, apoptosis, and mitochondrial–nuclear signaling—processes that are frequently hijacked by cancer cells.

From a strategic standpoint, mt-lncRNAs offer several distinct advantages as therapeutic targets: they are closely linked to mitochondrial bioenergetics, often display cancer-specific dysregulation, and target a critical vulnerability in tumor survival. By modulating mt-lncRNAs, it may be possible to directly impair mitochondrial fitness, sensitize cancer cells to chemotherapy, and overcome therapy resistance mechanisms that rely on mitochondrial adaptation.

However, significant challenges remain. Few mt-lncRNAs have well-defined functions. There are technical barriers to delivering nucleic-acid-based therapeutics across both cellular and mitochondrial membranes. It is also important to avoid off-target effects on normal mitochondrial function. Furthermore, most current approaches—such as antisense oligonucleotides, RNA interference, or mitochondrial-targeted delivery systems—remain at the preclinical or proof-of-concept stage.

Targeting mitochondrial genome-encoded lncRNAs is promising but immature. Success will rely on a deeper mechanistic understanding, better mitochondrial-specific delivery technologies, and rigorous validation in disease-relevant models. If these hurdles are overcome, mt-lncRNAs could become high-impact, precision targets for mitochondrial-driven cancers, including gynecological tumors.

## Figures and Tables

**Table 1:** Nuclear and mitochondrial lncRNAs: genetic origin, cellular localization, and functions.

		lncRNA Nuclear	lncRNA Mitochondrial
**Origin**	Genetic origin	Nuclear genome (nuclear DNA)	Mitochondrial genome (mtDNA)
	Cellular location	Nucleus, cytoplasm	Mitochondria (in the matrix or membranes)
**Functions**	Transcriptional regulation	Regulates nuclear gene expression	Regulates mitochondrial gene expression
	Epigenetic regulation	Recruits chromatin remodeling complexes	Regulate mtDNA (circular, histone-free)
	Metabolism regulation	Indirectly influences mitochondria through signaling	Directly affects OXPHOS, ATP production, mitophagy

**Table 2:** Mitochondrial lncRNAs involved in Gynecological Cancer.

Origin	Genomic Source	Examples	Function
Mitochondria-encoded	mtDNA	SncmtRNA, ASncmtRNA-1/2, lncND5/6, lncCytb/COX2	Retrograde stress signaling, apoptosis regulation, electron transport chain (ETC) modulation
Nuclear-encoded (mitochondria-localized)	Nuclear DNA	SAMMSON, LINC00116-related (mitoregulin lncRNA)	MAS regulation, mitochondrial translation, metabolic adaptation, ROS modulation

**Table 3:** Roles of Mitochondrial Anterograde Signaling Pathways in Gynecological Cancers.

Function	Impact of MAS Pathway
Mitochondrial biogenesis & dynamics	Nuclear transcription factors (PGC-1α, NRF1/2, TFAM) enhance mtDNA replication, OXPHOS protein expression, and fission/fusion balance, supporting proliferation and metastatic potential.
Mitochondrial metabolism regulation	Nuclear-encoded metabolic genes (c-Myc, HIF-1α, PPARs) modulate OXPHOS, TCA cycle enzymes, and fatty acid oxidation, promoting metabolic plasticity and survival under hypoxia or nutrient stress.
Stress adaptation & ROS buffering	Nuclear stress response genes (SOD2, NRF2, HSPs) induce antioxidant defenses and mitochondrial proteostasis, reducing ROS-induced damage and maintaining cell survival.
Apoptosis regulation	Nuclear BCL-2 family proteins modulate mitochondrial outer membrane permeabilization and cytochrome c release, promoting survival and chemoresistance.
Immune modulation	Reshapes TME, promotes immune evasion.
DNA damage & mitochondrial quality control	Nuclear p53, ATM/ATR signaling pathways regulate mitophagy, fission/fusion, and mtDNA repair, preserving mitochondrial integrity and promoting survival under chemotherapy stress.

**Table 4:** Role of Mitochondrial Retrograde Signaling Pathways in Gynecological Cancers.

Function	Impact of Retrograde Signaling Pathway
Metabolic reprogramming	Converts mitochondrial dysfunction into nuclear transcriptional programs, promoting glycolysis/OXPHOS flexibility and metabolic plasticity; supports tumor growth under hypoxic or nutrient-limited conditions.
Oxidative stress adaptation	Activates ROS-responsive pathways (NF-κB, MAPK/ERK, PI3K/AKT, STAT3), enhancing survival, DNA damage tolerance, and pro-inflammatory TME signaling.
Cell survival & proliferation	Alters Ca^2+^ signaling and stress-response transcription factors (Calcineurin → NFAT/CREB), supporting proliferation, survival, and apoptosis resistance.
Epithelial–Mesenchymal Transition (EMT) & metastasis	Activates HIF-1α, TGF-β, SNAIL/TWIST transcription programs, facilitating EMT, invasion, and metastatic dissemination, while modulating the immunosuppressive TME.
Cancer stemness maintenance	Engages NF-κB, Wnt/β-catenin, and STAT3 pathways to sustain cancer stem cell phenotypes, promoting tumor heterogeneity and therapy resistance.
Therapy adaptation/chemoresistance	Retrograde activation of p53, BCL-2 family, and NRF2 pathways enhances antioxidant defenses and anti-apoptotic signaling, enabling survival under chemotherapy.

**Table 5:** Comparative Roles of MAS and MRS in the regulation of tumor cell processes.

Process	MAS	MRS
Metabolic plasticity	Programs baseline metabolism	Rewires metabolism after stress
Glycolysis–OXPHOS switching	Establishes preferred metabolic phenotype	Triggers emergency metabolic switching
Hypoxia adaptation	Anticipatory hypoxia programming	ROS-driven adaptive hypoxic signaling
Chemoresistance	Intrinsic resistance programs	Acquired stress-induced resistance
Redox regulation	Baseline ROS tuning	Stress-driven ROS defense
Antioxidant programs	Intrinsic antioxidant capacity	Acute emergency antioxidant activation
Mitochondrial dynamics	Programmed DRP1/MFN2 balance	Damage-induced remodeling
Fusion/fission plasticity	Metabolic phenotype architecture	Mitochondrial quality control
ROS buffering	Steady-state redox control	Survival under oxidative stress
Immune Modulation	Indirect Immune Shaping via Metabolic Reprogramming Supports immune evasion via metabolic fitness	Direct via inflammatory signaling Strong activation (NF-κB, IFNs)
TME adaptation	Driven tumors adapt by becoming metabolically efficient and competitive	Creates inflammatory but often immunosuppressive TME (Suppression of CD8^+^ T cells; Polarization of TAMs toward M2 phenotype)

**Table 6:** Differential Roles of Key Transcription Factors/Proteins in MAS and MRS.

Molecules	MAS	MRS
**HIF-1α**	Pre-programs glycolysis and suppresses OXPHOS during planned hypoxia responses	Activated by mitochondrial ROS or ETC dysfunction → emergency metabolic switching
**NRF2**	Controlled by oncogenic pathways to establish an antioxidant baseline	Activated by mitochondrial stress → acute redox defense & survival signaling
**PGC-1α**	Drives mitochondrial biogenesis & metabolic phenotype specification	Induced after mitochondrial damage → compensatory mitochondrial repair/adaptation
**DRP1**	Structural remodeling to match metabolic demand	Stress-induced fragmentation and signal amplification
**MFN2**	Establishes mitochondrial fusion and OXPHOS efficiency	Modulates stress signaling and ER-mitochondria crosstalk
**PD-L1**	Its expression is a consequence of metabolic rewiring and hypoxia-driven adaptation	Its expression is directly linked to mitochondrial stress-induced inflammatory signaling pathways

**Table 7:** Classes of mitochondrial lncRNAs involved in bidirectional signaling pathways in Gynecological Cancers.

lncRNA	MAS or MRS	Key Molecular Markers Linked	Functional Impact	Cancer Context (Evidence)
**SncmtRNA**	**MRS**	ROS, ΔΨm, apoptosis regulators	Associated with proliferative state; altered during transformation	Cervical cancer
**ASncmtRNA-1**	**MRS**	Caspases, mitochondrial stress, NF-κB (indirect)	Downregulated in tumors; knockdown induces apoptosis	Cervical cancer
**ASncmtRNA-2**	**MRS**	ROS, apoptosis pathways	Processed into small RNAs; inhibition reduces tumor cell viability	Cervical cancer
**lncND5/lncND6**	**Likely MRS-associated**	Complex I (ND5/ND6), ROS, ETC dysfunction	Proposed regulation of respiratory chain gene expression under stress	Reported in mitochondrial transcriptome analyses (limited gynecologic-specific data)
**lncCytb/lncCOX2**	**Likely MAS/MRS interface**	Complex III (Cytb), Complex IV (COX2), OXPHOS efficiency	Potential modulation of ETC assembly and mitochondrial bioenergetics	Identified in mitochondrial transcript studies
**SAMMSON**	**MAS**	p32 (C1QBP), mitochondrial ribosome, OXPHOS proteins	Regulates mitochondrial translation; supports metabolic fitness and survival	Ovarian cancer
**LINC00116-related (mitoregulin transcript)**	**MAS**	OXPHOS complexes, mitochondrial membrane potential	Encodes a micropeptide regulating mitochondrial respiration and bioenergetics	Ovarian cancer
**H19**	**MAS**	PGC-1α, NRF1/2, TFAM, OXPHOS complex proteins, NAD^+^/NADH	Promotion of mitochondrial biogenesis, oxidative metabolism, chemoresistance	Ovarian (HGSOC)

**Table 8:** Targeting Strategy of Mitochondrial lncRNAs in Gynecological Cancers.

Targeting Strategy	lncRNA	Genome Origin	Signaling Direction	Gynecologic Cancers
ASOs	ASncmtRNA-1/2	Mitochondrial	Retrograde	Cervical, ovarian
ASO, OXPHOS inhibitors	lncND5	Mitochondrial	Retrograde	Ovarian
ASOs, chemo-combination	SAMMSON	Nuclear	Anterograde/ bidirectional	Ovarian, cervical
siRNA, nanoparticles	LINC00152	Nuclear	Anterograde	Ovarian
ASOs, siRNA, transcriptional modulators	H19	Nuclear	Anterograde	Cervical, ovarian, endometrial
ASOs, RNA modulation	GAS5 (mitochondrial pool)	Nuclear	bidirectional	Ovarian, cervical, endometrial

## Data Availability

No new data were created or analyzed in this study. Data sharing is not applicable to this article.
